# A Definite Step Toward Clinical Implementation of AI‐Assisted Rapid On‐Site Evaluation During EUS‐TA

**DOI:** 10.1111/den.70137

**Published:** 2026-03-13

**Authors:** Yuki Fujii, Kazuyuki Matsumoto, Motoyuki Otsuka

**Affiliations:** ^1^ Department of Endoscopy Okayama University Hospital Okayama Japan; ^2^ Department of Gastroenterology and Hepatology Okayama University Hospital Okayama Japan

**Keywords:** artificial intelligence, endoscopic ultrasound‐guided fine needle aspiration, pancreatic cancer, rapid on‐site evaluation

1

Rapid on‐site evaluation (ROSE) has played an important role in improving diagnostic performance of endoscopic‐ultrasound‐guided tissue acquisition (EUS‐TA) for pancreatic masses [1, 2, 3]. The diagnostic accuracies for pancreatic cancer with or without ROSE do not differ with the use of EUS‐guided fine‐needle biopsy (EUS‐FNB) needles according to results mainly obtained from high‐volume centers [4]. Whether similar outcomes can be achieved in institutions with less procedural experience remains unclear. ROSE may increase diagnostic accuracy in diagnostically difficult cases requiring repeat EUS‐TA and may help avoid unnecessary additional punctures by enabling the real‐time assessment of specimen adequacy [5, 6]. Despite these benefits, the global shortage of cytopathologists, along with the associated human resource and economic burdens associated with ROSE, has limited the widespread use of ROSE. As such, artificial‐intelligence‐assisted ROSE (AI‐ROSE) is increasingly attracting attention as a solution to these challenges and as a tool to standardize EUS‐TA among institutions. Although several AI‐ROSE systems have been developed, key issues regarding the diagnostic accuracy, processing speed, and real‐world clinical applicability remain unresolved [7, 8, 9].

In this issue of *Digestive Endoscopy*, Ashida et al. provide a comprehensive evaluation of an AI‐ROSE system for pancreatic EUS‐TA [10]. The researchers advanced the system beyond proof‐of‐concept: the system rapidly achieved high diagnostic accuracy, distinguishing the system from previous AI‐ROSE methods. First, the diagnostic framework closely captures how cytopathological evaluations are performed in clinical practice. Rather than using a simple binary classification, the authors designed a five‐tier system consolidated into three clinically meaningful categories, incorporating the diagnostic uncertainty associated with borderline or atypical lesions. This approach represents methodological advance that aligns well with real‐world decision‐making. In an independent validation cohort, the system accurately diagnosed 95.1% of malignant lesions. Second, the authors directly compared the diagnostic accuracy as well as evaluation time between AI‐ROSE and human evaluators, including endoscopists with different levels of experience and cytopathologists. AI‐ROSE outperformed all human evaluators in terms of diagnostic accuracy, and 120 clusters were assessed in approximately 6 s, which was much faster than the humans. This speed system directly supports real‐time decision‐making during EUS‐TA.

The main contribution of AI‐ROSE is the ability to stabilize cytological assessment and reduce interobserver variability without additional human effort. Even experienced cytopathologists may be affected by fatigue, and evaluations are affected by differences in human experience, which impact diagnostic consistency. In this context, the segmentation‐based approach provides visual information that shows which cell populations correspond to specific diagnostic categories. This visualization enhances diagnostic objectivity and offers educational value for less‐experienced endoscopists and cytopathology residents. In addition to these advantages, methodological considerations in development of AI‐ROSE system are also important. Our previous study suggested that the appropriate selection of data augmentation strategies may contribute to improving diagnostic performance in AI‐ROSE system development, while whole‐slide image‐based evaluation was employed to reduce selection bias arising from subjective field selection [9]. Crucially, AI‐ROSE is not a replacement for the cytopathologists responsible for the final diagnosis. Instead, AI‐ROSE functions as a supportive tool, providing expert‐level guidance without time or location constraints. In institutions without on‐site cytopathologists, AI‐ROSE may assist endoscopists in decision‐making, increase workflow efficiency, and contribute to standardizing EUS‐TA quality.

Despite steady progress in AI‐ROSE research, several challenges remain before the widespread clinical implementation of AI‐ROSE. Unlike computer‐aided diagnosis (CAD) for endoscopic imaging, cytological slide data used for training and validation are strongly influenced by environmental factors. Smear techniques, staining protocols, microscope settings, observation conditions, and specimen quality widely vary among institutions. Ashida et al.'s study used smears prepared at a single institution, and large‐scale multicenter validation across heterogeneous institutional environments is required to establish broader generalizability. These differences may affect the AI‐ROSE performance. Even subtle factors such as staining time or deterioration of the specimens used for training should not be overlooked when using AI‐ROSE. Therefore, specimen preparation must be strictly standardize for the clinical implementation of AI‐ROSE. In addition, the entire workflow, including microscopes, analysis computers, and hospital information systems, must be integrated. For these reasons, the clinical implementation of AI‐ROSE may be more challenging than that of the already‐in‐use endoscopic image‐based CAD systems in clinics. In addition to technical performance, the cost of implementation may represent a potential barrier to the widespread adoption of AI‐ROSE in real‐world clinical settings. However, as digital cytology and cloud‐based platforms continue to expand, software‐driven AI systems may become increasingly accessible, even in institutions without on‐site cytopathologists. Furthermore, explainability, clarifying how and why AI reaches a particular decision, is critical for the clinical acceptance of AI‐ROSE. Clear visualization tools such as heat maps and segmentation overlays will become increasingly important for describing the process of AI‐based decision‐making. Finally, most studies have focused on pancreatic cancer, and data on other pancreatic tumors such as neuroendocrine or solid pseudopapillary neoplasms remain limited. Further analyses that reflect real‐world clinical diversity are required.

Ashida et al. achieved a milestone, indicating that AI‐ROSE has transitioned from the research phase to real‐world clinical applications. The authors advance AI‐ROSE toward clinical feasibility using rigorous methods, evaluations based on actual EUS‐TA practice, and a focus on real‐time performance. Future efforts should focus on confirming the reproducibility in diverse clinical settings and clarifying how AI‐ROSE supports endoscopist decision‐making in routine practice. Prospective multicenter studies are required to validate the clinical utility of AI‐ROSE under standardized specimen preparation protocols across multiple institutions.

## Author Contributions

Y.F. conceived the topic and drafted the initial manuscript. K.M. and M.O. provided critical revisions and editorial guidance.

## Funding

The authors have nothing to report.

## Conflicts of Interest

The authors declare no conflicts of interest.

## Linked Article

This article is linked to Ashida et al. papers. To view this article, visit http://doi.org/10.1111/den.70081.
